# Real sweating in a virtual stress environment: Investigation of the stress reactivity in people with primary focal hyperhidrosis

**DOI:** 10.1371/journal.pone.0272247

**Published:** 2022-08-02

**Authors:** Andrea B. Schote, Katharina Dietrich, Adrian E. Linden, Inga Dzionsko, Laura De Los Angeles Molano Moreno, Ulrike Winnikes, Patrick Zimmer, Gregor Domes, Jobst Meyer

**Affiliations:** 1 Department of Neurobehavioral Genetics, Institute of Psychobiology, University of Trier, Johanniterufer, Trier, Germany; 2 Department of Biological and Clinical Psychology, University of Trier, Johanniterufer, Trier, Germany; Nippon Medical School, JAPAN

## Abstract

**Background:**

Hyperhidrosis (excessive sweating, OMIM %114110) is a complex disorder with multifactorial causes. Emotional strains and social stress increase symptoms and lead to a vicious circle. Previously, we showed significantly higher depression scores, and normal cortisol awakening responses in patients with primary focal hyperhidrosis (PFH). Stress reactivity in response to a (virtual) Trier Social Stress Test (TSST-VR) has not been studied so far. Therefore, we measured sweat secretion, salivary cortisol and alpha amylase (sAA) concentrations, and subjective stress ratings in affected and non-affected subjects in response to a TSST-VR.

**Method:**

In this pilot study, we conducted TSST-VRs and performed general linear models with repeated measurements for salivary cortisol and sAA levels, heart rate, axillary sweat and subjective stress ratings for two groups (diagnosed PFH (n = 11), healthy controls (n = 16)).

**Results:**

PFH patients showed significantly heightened sweat secretion over time compared to controls (*p* = 0.006), with highest quantities during the TSST-VR. In both groups, sweating (*p* < 0.001), maximum cortisol levels (*p* = 0.002), feelings of stress (*p* < 0.001), and heart rate (*p* < 0.001) but not sAA (*p* = 0.068) increased significantly in response to the TSST-VR. However, no differences were detected in subjective ratings, cortisol concentrations and heart rate between PFH patients and controls (*p*_all_ > 0.131).

**Conclusion:**

Patients with diagnosed PFH showed stress-induced higher sweat secretion compared to healthy controls but did not differ in the stress reactivity with regard to endocrine or subjective markers. This pilot study is in need of replication to elucidate the role of the sympathetic nervous system as a potential pathway involved in the stress-induced emotional sweating of PFH patients.

## Introduction

Hyperhidrosis (HH), i.e. profuse sweating due to heat, mental or emotional stress, puts a strain on people in a society, which deems excess perspiration unhygienic and unacceptable [1]. Hyperhidrotics suffer from abnormal degrees of perspiration at various body sites. This leads to soaked clothing, axillary sweat stains, cold and wet handshakes and many more manifestations that result in ‘occupational, emotional, psychological, social, and physical impairment’ [2], and ultimately, even self-imposed social isolation [3]. Often, excess sweating can be explained as a secondary, concomitant feature of conditions such as overweight, diabetes, or viral infections. However, in addition to such origins, there exists a primary, inherited form of HH as well. Primary focal hyperhidrosis (PFH) has a prevalence of 0.6 to 3.2% [4], however with large variation in different ethnic groups [2,5–9]. PFH is characterized by excessive sweating at particular body regions, mainly palms, plants, and armpits, and has its onset typically in adolescence [10–13]. PFH is a disorder *per se* and not caused by another medical condition, nor is it a side effect of medications. As many similar idiopathic disorders, PFH was considered a purely psychiatric condition for a long time, a notion reinforced by the association of perspiration with anxiety [14]. In the ICD-10, localized hyperhidrosis is characterized as R61.0. This means that although objective measurements of the absolute sweat secretion can be performed, the impact of hyperhidrosis and its diagnosis highly depend on the individual perception, and hence the psychological strain of the affected person. A putative cause of PFH might be the increased sympathetic activity of the autonomic nervous system, resulting in the innervation of sweat glands by postganglionic sympathetic fibres or the activation via circulating catecholamines [13,15]. Mental stimuli [16] and stressful situations [17] can trigger hyperhidrotic flare-ups. The increased sensitivity of sweat centres located in the mesencephalon, in the medulla oblongata and in the lateral column might be a reason for the increased emotional sweating [5].

Processing mental and emotional stress stimuli is presumed to follow complex mechanisms. Under stressful conditions, the fight-or-flight response, crucial to survival and the maintenance of homeostasis, triggers responses on a behavioural as well as physiological level via the neuroendocrine, immune and sympathetic nervous system, which involve (in order of appearance) 1) the activation of the sympatho-adrenal-medullary (SAM) system resulting in the release of (predominantly) catecholamines upregulating heart rate, blood pressure, salivary alpha amylase (sAA) and respiration, 2) the activation of the hypothalamic-pituitary-adrenocortical (HPA) axis upregulating glucocorticoid levels (e.g. cortisol), and 3) the activation of the immune system releasing cytokines, free radicals and prostaglandins [18]. Upon activation of the systemic HPA axis, corticotropin releasing hormone (CRH) and arginine-vasopressin in the hypothalamus trigger the production of adrenocorticotropic hormone (ACTH) in the anterior pituitary gland. This stimulates the adrenal glands to secrete the predominant stress hormone cortisol, which acts upon hypothalamus, pituitary gland, and hippocampus [19,20]. Additionally, a cutaneous equivalent to the HPA axis exists, which mediates responses to mechanical and thermal disturbances [21] as well as UV radiation [22] prior to involvement of the central nervous system. Atypical from the default sympathetic process, the dominant neurotransmitter in psychological sweat responses is acetylcholine (ACh). The release of ACh by sympathetic and not parasympathetic nerves, instead of noradrenaline, might be due to the skin’s low grade of parasympathetic innervation [23].

The cognitive appraisal of the stressfulness of a situation varies between individuals and can best be observed in studies using highly standardized laboratory stressors (e.g. the Socially-Evaluated Cold Pressor Test, SECPT, [24]; or Maastricht Acute Stress Test, MAST, [25]). Among these protocols, the Trier Social Stress Test (TSST) [26] has become widely used in psychobiological stress research as it has been proven to evoke robust endocrine and cardiovascular responses in most participants. The TSST mainly consists of a short job interview and a mental arithmetic test in front of an audience of three people. It thus implements two main factors for robust stress-induced HPA-axis activation: social evaluative-threat and uncontrollability [27]. An effective adaptation of the original TSST is the virtual-reality TSST (TSST-VR). VR applications have been previously used in many fields of psychology especially in psychotherapies to create scenarios that are cumbersome to implement on other ways [28–31]. In 2007, the TSST was adapted for the first time in a virtual environment. Kelley and colleagues demonstrated the potential of studying the effects of socially evaluative stress induction in VR with respect to subjective and neuroendocrine stress reactivity [32]. Since then, there have been several further developments of this paradigm and the effectiveness of the TSST-VR in terms of psychological and autonomous stress responses was shown [33,34]. Recently, the TSST-VR has been used in combination with eye tracking to access stress-related gaze behavior [29], showing the potential of the TSST_VR for investigating the role of acute stress in modulating social cognitive functioning. The use of TSST-VR significantly reduces the need of extensively trained judges, allows a maximum of experimental control, increases standardization of the procedure and most importantly leads to similar physiological and psychological stress reactions as in an *in vivo* situation [34–36].

As patients with PFH suffer especially in situations, which are associated with social stress [19], the investigation of this link seems to be interesting, however rarely looked at. In a recent review, a conceptual model of psychological sweating shows that stress increases cholinergic neural innervations of eccrine sweat glands but found no evidence that the elevation of circulatory glucocorticoid release by the HPA axis directly modulates sweat glands activity [18]. Since there is no research on the stress reactivity of patients with PFH in a standardized stress test recording the amount of sweat, salivary cortisol, sAA and heart rate so far, we aimed to compare two groups (PFH patients vs. healthy controls) and test the following hypotheses in a pilot study: I) PFH patients report higher subjective stress ratings and higher cortisol levels in response to the stress test compared to the control subjects. II) PFH patients respond with increased SAM activation reflected in sAA levels but do not differ from healthy control subjects in terms of heart rate. III) Due to the suggested hyperactivity of the sympathetic system, a higher perspiration is expected in PFH patients compared to healthy control subjects.

## Material and methods

### Ethics statement

The study was approved by the Ethics Committee of the University of Trier (ethical identification number 76/2018) and conducted in accordance with The Code of Ethics of the World Medical Association (Declaration of Helsinki) for experiments involving humans and the American Psychological Associations’ Ethical Principles of Psychologists and Code of Conduct. All participants gave a written informed consent that they took part in the study voluntarily.

### Participants and design

The experimental design comprises one between-subjects factor with two levels. Participants were either diagnosed patients with PFH or healthy control subjects. In both groups, participants performed the TSST in a virtual environment.

Participants were recruited via advertisements on the campus of the University of Trier, in surrounding general and dermatological practices, in pharmacies and schools in the Trier district. Additionally, a call was placed in local newspapers and standardized e-mails were sent via the distributor of the University of Trier, the University of Luxembourg and the Trier University of Applied Sciences.

**Fig 1:**
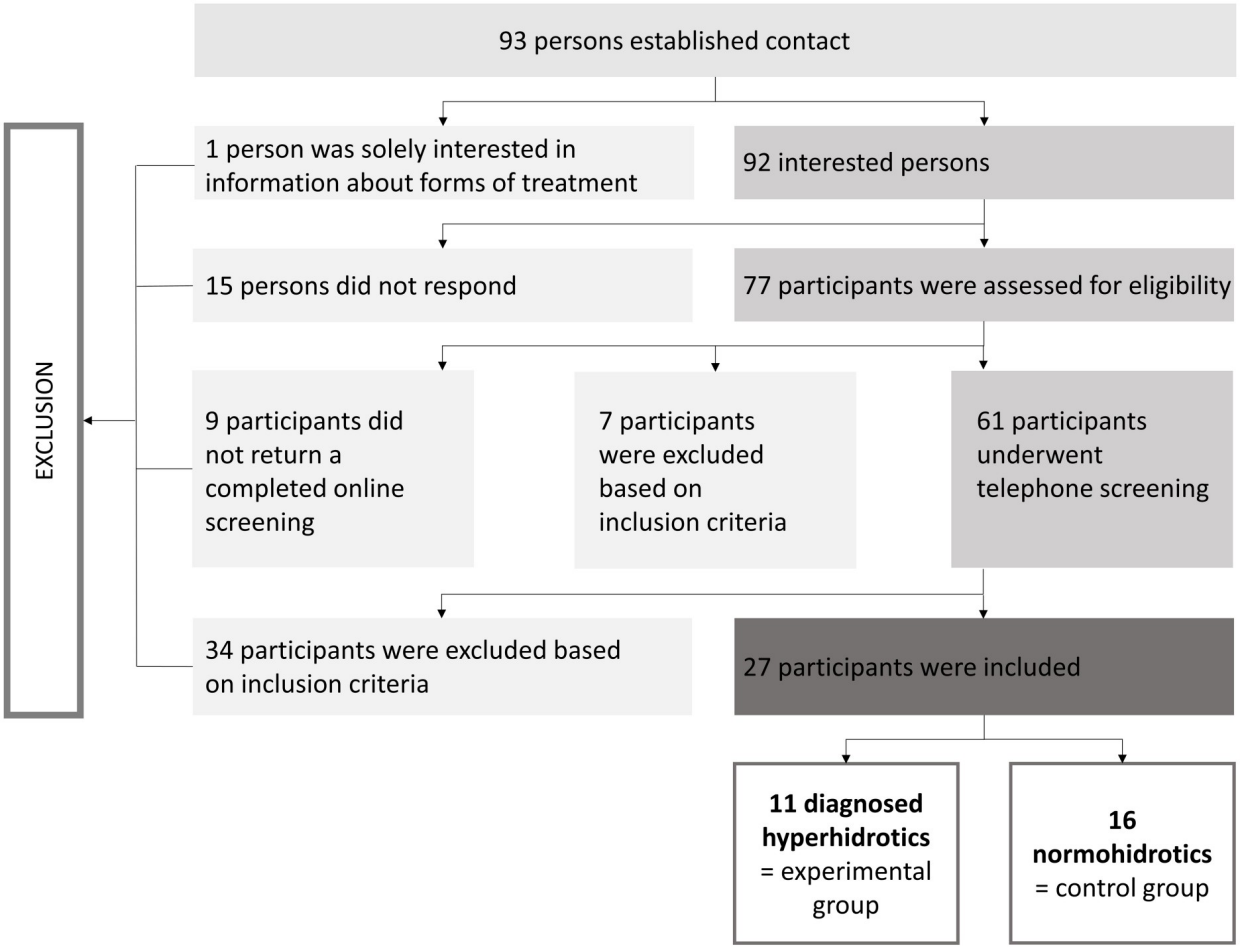
Flowchart of the sample included in the pilot study. Illustration of the strategy and different stages of recruitment. From initially 93 interested people, 27 subjects were included in the study.

During the survey period (October 2018 to April 2019), 93 people made contact as a result of the recruitment strategies. Of these, 92 people expressed interest in the study, with 1 person just wanting to learn about possible forms of therapy. Of these interested ones, 77 people received the online screening form (https://osf.io/hn48m/?view_only=2c0a4141472e4212ba6032593911952d), in which general questions concerning sex, age, height, weight, physical and psychological wellbeing were asked. From the initial 77 people, 15 no longer responded; 9 did not return a completed online screening, and seven had to be excluded based on the following exclusion criteria. These were: wearing glasses that cannot be left out or replaced by contact lenses for the experiment, smoking more than five cigarettes per day, drinking regularly more than one litre of wine or three glasses of beer, consuming drugs [37]. As a result, a telephone screening (https://osf.io/hn48m/?view_only=2c0a4141472e4212ba6032593911952d) was carried out with 61 people to validate the criteria of the online screening form. The following exclusion criteria were applied: participation on previous stress tests or VR experiments, working night shifts, BMI below 19 and above 25 kg/m^2^, acute or chronic somatic or psychiatric disease (incl. allergies, asthma, epilepsy, thyroid disorders, cardiovascular disorders, neurodermatitis, neurological disorders, diabetes), regular intake of medication, especially endocrine active drugs (cortisone or alike) or antihistaminica, psychotherapeutic treatment in the last 12 months, menopause [37]. Finally, 27 people could be included and assigned to two groups: 11 diagnosed patients with PFH and 16 controls (Fig 1). The participants with PFH were stating that they had received diagnosis from healthcare and in addition answered the hyperhidrosis severity scale (HDSS) [38]. All participants answered the hyperhidrosis impact questionnaire (HHIQ) [39]. For 36.4% of the participants with PFH “[the] sweating is tolerable but sometimes interferes with [the] daily activities”, for 36.4% “[the] sweating is barely tolerable and frequently interferes with [the] daily activities” and for 9.1% “[the] sweating is intolerable and always interferes with [the] daily activities”, from 2 participants the answers were missing. While the group of diagnosed PFH patients consisted of 4 women and 7 men (*M*_*age*_ = 36.1; *SD* = 17.6; age range = 20–65), the control group included 9 men and 7 women (*M*_*age*_ = 33.3; *SD* = 16.1; age range = 19–66).

Prior to the start of the testing session, participants were asked to refrain from physical exercise and consumption of alcoholic or caffeinated beverages at least 24h and to abstain from consuming anything but water in the preceding two hours of the experiment. Additionally, participants were asked not to use deodorants or antiperspirants on the day of the experiment and to wear casual clothing.

### Apparatus

**Fig 2:**
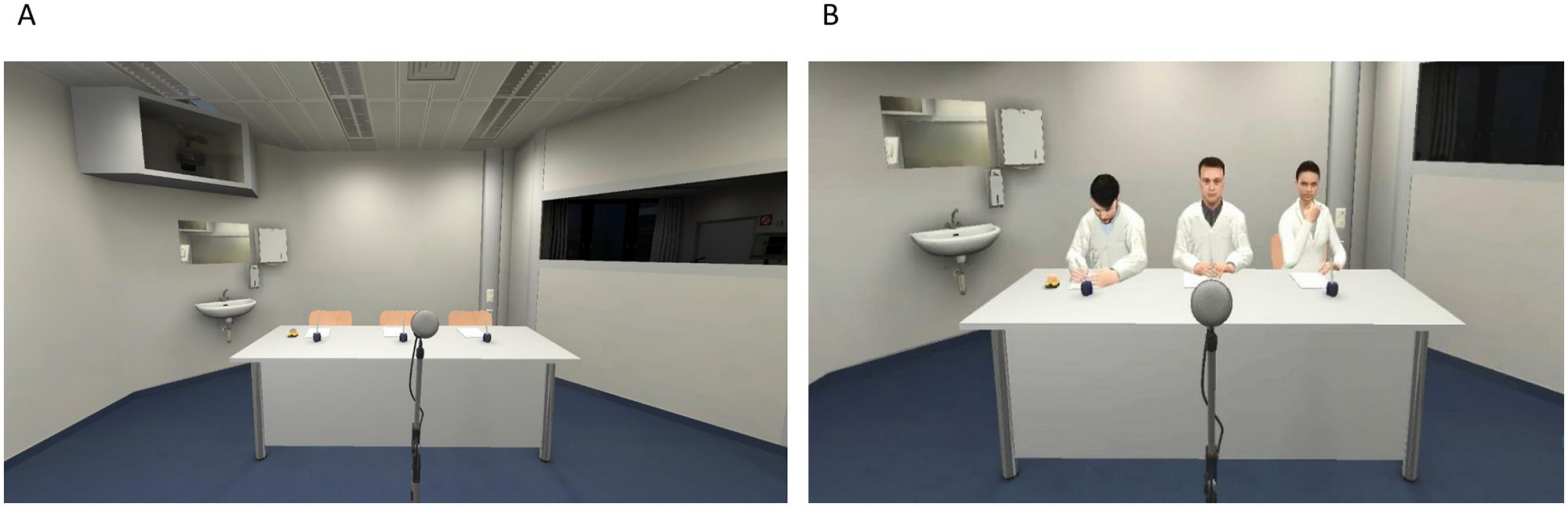
Depiction of the virtual environment. Participant’s view during the TSST a) before Jury enters the room and b) with Jury being present in the setting.

In previous studies, the VR application was created and tested [29,34,35]. The virtual environment was generated using the Steam Source engine (Valve Corporation, Bellevue, Washington, USA), interfaced by the VR simulation software CyberSession 5.6 (VTPlus GmbH, Würzburg, Germany), and operated via the CSRemote IOS app running on an Apple IPad Air. The experiment ran on a desktop computer (Intel Core i7 4790K @ 4 Ghz, 16 GB Dual-Channel DDR3 RAM @ 3900 Mhz, NVidia Geforce GTX 980Ti with 6 GB of GDDR5 VRAM). A Head-Mounted Display (HMD; Oculus Rift DK2, Oculus VR LLC, Menlo Park, CA, USA; resolution: 1920 x 1080 [960 x 1080 pixels per eye]; field of view: 100°) with integrated head-tracking was used for VR simulation. Sound was presented via headphones. The virtual environment in the simulation was designed to closely resemble one specific VR laboratory experimentation room. It shares its distinctive features like a large one-way-mirror on the right, a sink in the left corner as well as a white desk with three chairs and a microphone in the middle of the room. Fig 2 shows the participants’ view during the TSST. More screenshots of the virtual environment are shown in the supporting information (S1 Fig). Further technical specifications can be found in the supplementary methods published by Zimmer *et al*. [34]. Heart rate (HR) was recorded using an ANS Recorder flex mobile ECG device (Neurocor Ltd. & Co. KG, Trier, Germany). IBI files were exported using the most artifact-free ECG derivation of the three possible alternatives and entered into ARTiiFACT [40] for automatic artifact detection and correction using cubic spline interpolation [41]. If necessary, automatically corrected files were reintroduced in ARTiiFACT and manually corrected after having undergone visual inspection.

### Measures

To assess the subjective stress response, the participants rated a single question “How stressed do you feel at the moment?” at -35, -20, +15, +40 and +60 minutes (in reference to TSST onset) on a visual analogue scale (VAS; range from 0—not at all to 100—very much, 10 cm length) [42].

**Fig 3:**
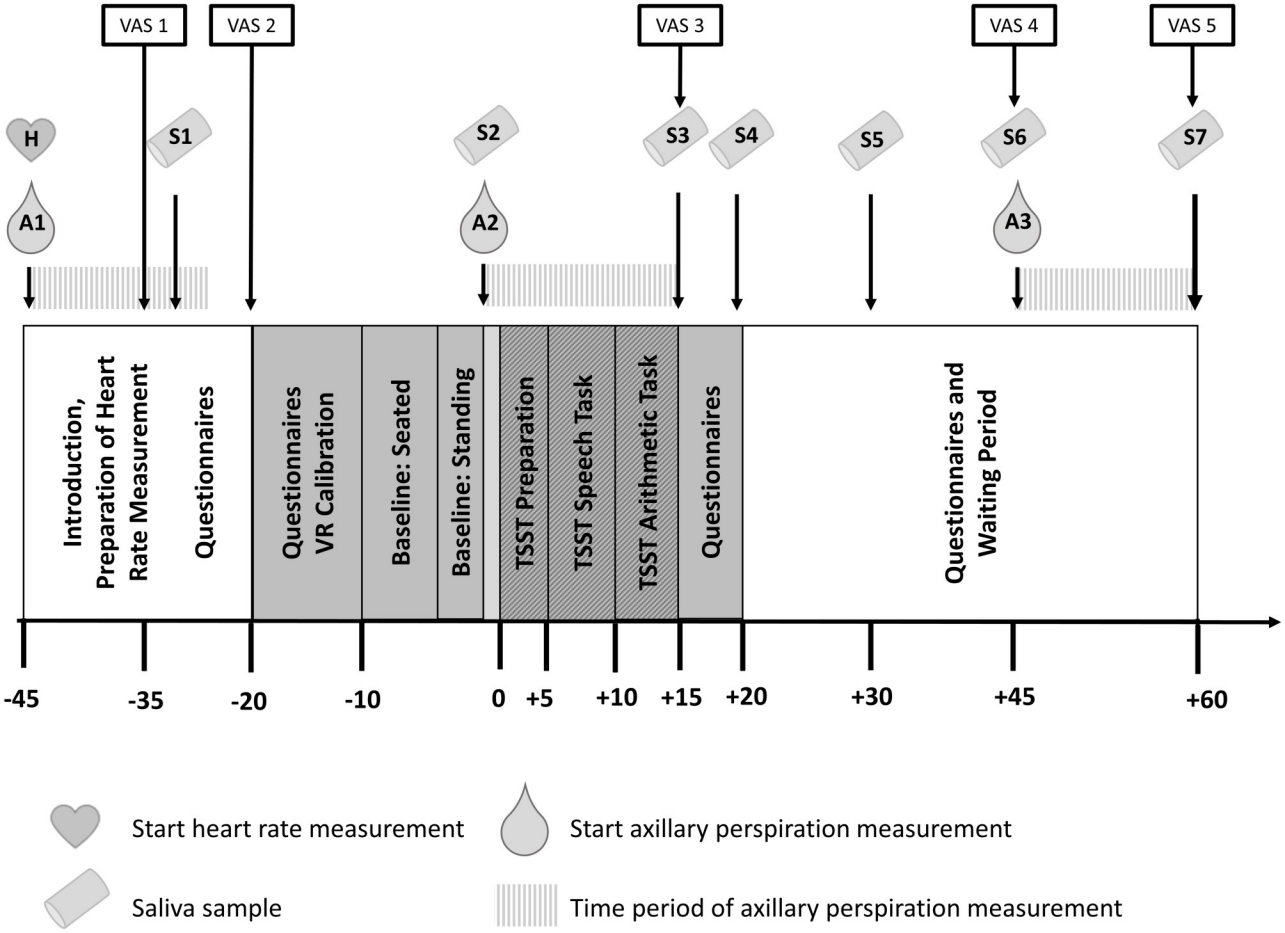
Experimental procedure. Experimental phases and time of assessment of subjective stress ratings (VAS), saliva samples (S) and sweat measures are depicted. Procedures in the preparation room are shown in white; procedures in the VR laboratory have been marked in grey. Hatched patterns represent the components of the TSST.

At seven time points (Fig 3), participants were asked to give saliva samples by using Salivettes (Sarstedt, Nümbrecht, Germany). Samples were stored at– 20 °C until biochemical analysis by the University Laboratory to determine concentrations of free salivary cortisol and sAA. For cortisol analysis, a time-resolved fluorescence immunoassay [43] was used. 100 μl of saliva were used for duplicate analysis (50 μl per well). The Intra-assay coefficient of variation ranged between 4.0% and 6.7% and the corresponding inter-assay coefficients of variation were between 7.1% -9.0%.

For sAA analysis, the chromogenic molecule 2-Chloro-4-nitrophenyl-a-D-maltotrioside was used [44]. Saliva was diluted 1:200 with assay diluent. 16 μl of the diluted saliva were used for duplicate analysis (8 μl per well). The intra-assay coefficient of variation was between 2.8% and 6.3%, and the corresponding inter-assay coefficients of variation were between 5.5%–7.6%.

HR was measured throughout the whole experiment using ECG electrodes that were placed immediately after arrival and removed close to the end before leaving the laboratory (Fig 3).

Axillary sweat secretion was gravimetrically measured before, during and after the TSST-VR as indicated in Fig 3. For this purpose, weighed gauze swabs (10 x 10 cm, Nobamed Paul Danz AG, Wetter, Germany) were fixed under both armpits using tape, removed after 15 minutes, placed into a zipped plastic back and weighed after 15 minutes on a precision balance [45]. For each armpit the sweat rate was calculated as the difference between full (swab weight after 15 minutes carrying) and empty (swab weight before carrying). For the group comparisons the mean value of sweat rates of both armpits were used. To avoid gender-dependent effects, all interaction with the participants was done by the same-sex experimenter.

### Procedure

Experimental sessions were scheduled to start at either 3 p.m. or 5 p.m. to control for the circadian secretion rhythm of cortisol [46]. Upon arrival, participants were greeted by the experimenter and an assistant and informed about the following procedures before declaring their consent. After application of the ECG device and fixation of the first gauze swabs under the armpits, participants were asked to fill out the first VAS and to give the first saliva sample. Afterwards, participants were brought to the VR laboratory room. Humidity and temperature were measured and documented before each experimental setting. The temperature in the preparation room was on average 20.3 °C (SEM 0.2) and in the VR laboratory 22.4 °C (SEM 0.4). Humidity in the preparation room was on average 37.1 (SEM 1.1) and in the VR laboratory 32.0 (SEM 1.3). After filling out a second VAS, participants put on the HMD and headphones. They subsequently had ten minutes to familiarize themselves with their virtual surroundings. After this baseline phase, the experimenter fixated the second set of gauze swabs under the armpits of the participant and the second saliva sample was given. Then, participants received instructions on the following task via head phones and in writing on the screen.

Participants were told that they would have three minutes to prepare for a job interview in front of a panel of judges. Afterwards, three virtual judges entered the room, took their places behind a desk and informed the participants that the preparation period was now beginning (Fig 3). Apart from the shorter preparation time and the need to prepare the job interview without being able to take notes, TSST procedures were directly adapted from the original paradigm [26]. During the tasks, the virtual judges were controlled by the experimenter who triggered pre-recorded follow-up questions and instructions. After the arithmetic task, the virtual judges stood up and left before the screen turned black. Participants were then assisted in taking off the HMD and asked to remain standing while giving the third saliva sample and filling out the VAS. After completion, participants were led back to the preparation room where they periodically gave additional saliva samples and answered questionnaires. Fixation of the third gauze swabs was paired with the sixth saliva sample. The experimental procedure ended 60 minutes after the beginning of the stress induction when participants gave the last saliva sample and questionnaires before being debriefed and compensated (Fig 3).

### Statistical analysis

All analyses were carried out in SPSS for Windows (Version 25) based on the protocol of Zimmer *et al*. [34]. Prior to the main analysis, data was tested for normal distribution and homoscedasticity using the Kolmogorov Smirnov test and Levene’s test, respectively. Group differences in sample characteristics, including age, weight, height, and BMI were analysed using unpaired *t* tests and gender distribution was compared between groups using a χ^2^ test. Results are shown in S1 Table. The data derived from the HHIQ were evaluated using descriptive statistics. To test for effects of group (PFH vs. healthy controls) and time over the course of the experiment (as a repeated-measures factor) on subjective and physiological stress markers (the latter comprised salivary cortisol and sAA, heart rate, and axillar perspiration), we conducted mixed repeated-measures ANOVAs. The first time point (-35) was excluded from the final analyses of salivary cortisol and sAA, as we could not control for the strong individual anticipation effect at this time point in our model. Greenhouse-Geisser corrections were applied in cases where the assumption of sphericity was violated (indicated by significant Mauchly’s tests), *ε*- and corrected *p*-values are reported, accordingly. In cases of significant group x time interactions, group differences were additionally analysed at each time point using Bonferroni-corrected pairwise comparisons or, in case the assumption of normal distribution was violated, Mann Whitney *U* tests. An overview of group’s scores on all dependent variables (*M* and *SD*) and their differences at each time point can be found in S2–S6 Tables. Significance level was set at *p* < 0.05 and effect sizes are reported as η_*p*_^*2*^. Dataset and codebook are available at the link https://osf.io/hn48m/?view_only=2c0a4141472e4212ba6032593911952d.

## Results

### Hyperhidrosis impact questionnaire

There were no significant gender (χ^2^ = 0.147, *p* = 0.701) and age (*t*(25) = -0.424, *p* = 0.675) differences between the groups (S1 Table). In the PFH group, 27.3% of the subjects reported the onset of their hyperhidrosis at an age between 12 and 17 years, 27.3% were over 17 years old, 27.3% were between 6 and 11 years and 18.2% were under the age of 6 when their excessive sweating began. The body regions most frequently affected in the PFH group were the armpits and palms with each 36.4% followed by plants and face with each 9.1%. One participant did not answer that question. 18.2% of the participants in the PFH group reported significant emotional impairment, small or moderate emotional burden was found in each 36.4% of the participants, 9.1% were not impaired by their hyperhidrosis. The reported sweat spot size in the PFH group was in 9.1% more than twice as big as the own palm, in 27.3% twice as big as the own palm, in 45.5% as big as the own palm and in 9.1% smaller than the own palm.

Stress was reported by 90.9% of participants in the PFH group but only 43.3% of the participants in the control group as a situation that trigger excessive sweating. The most commonly reported accompanying phenomenon in both group were cold feet (PFH group 63.6%, control group 43.8) and cold hands (PFH group 54.5%, control group 50%).

### Self-reported stress

A 2 (PFH vs. healthy controls) x 5 (time) repeated-measures ANOVA was conducted to analyse effects of the stressor on subjective stress ratings measured with the item “How stressed do you feel at the moment?” on a VAS scale from 0 to 100. Due to missing data of one PFH patient, this analysis was applied to a sample of *N* = 26. The results revealed a significant main effect of the factor time (*F*(2.589, 62.132) = 22.038, *ε* = 0.647, *p* < 0.001, *η*_*p*_^*2*^ = 0.479, 95% CI [15.170; 27.899]), indicating significantly increased feelings of stress in response to the stressor, reaching its peak at the end of the TSST-VR before returning to baseline levels in the recovery phase. No significant group x time interaction effect was found (*F*(2.589, 62.132) = 0.517, *ε* = 0.647, *p* = 0.646), indicating similar subjective stress responses of PFH patients and healthy controls.

### Free salivary cortisol

**Fig 4:**
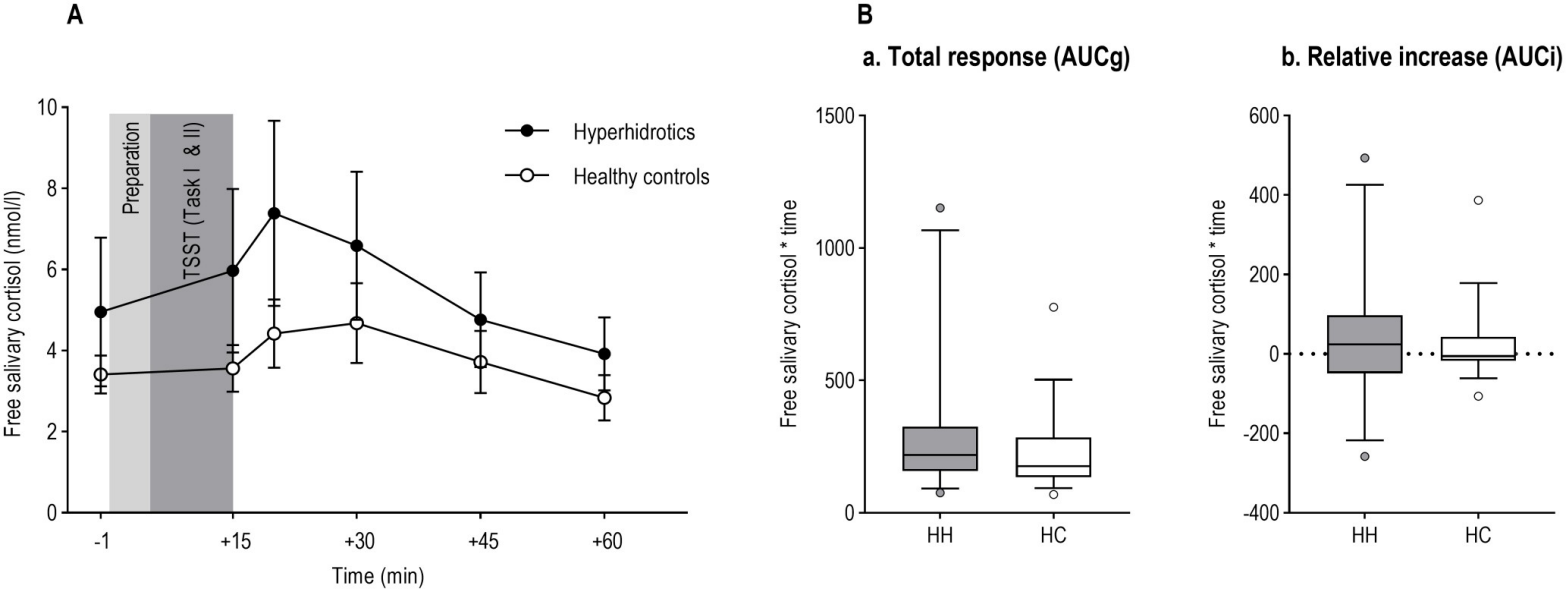
Analyses of salivary cortisol. (A) Free salivary cortisol (nmol/l) in response to the stress condition over time. Preparation and TSST (Task I & II) represent the respective phases of the TSST. Error bars denote standard errors. (B) Boxplots showing Area under the Curve (AUC) values calculated for free salivary cortisol in response to the stress for patients with PFH (HH) and healthy controls (HC). (a) Area under the Curve with respect to ground (AUCg). (b) Area under the Curve with respect to increase (AUCi). Whiskers denote 10^th^ and 90^th^ percentile.

We conducted a 2 (PFH vs. healthy controls) x 6 (time) repeated-measures ANOVA to test for a stress-related increase in free salivary cortisol and potential group differences of the latter (S2 Table). Results of the analysis revealed a significant main effect of the factor time (*F*(2.166, 54.153) = 6.901, *ε* = 0.433, *p* = 0.002, *η*_*p*_^*2*^ = 0.216, 95% CI [3.099; 6.271]), indicating an increase in salivary cortisol over time with its maximum at 20 minutes post onset of the stressor. The group x time interaction effect did not reach significance (*F*(2.166, 54.153) = 1.096, *ε* = 0.433, *p* = 0.345), indicating that groups did not differ regarding their HPA reactivity in terms of secretion of salivary cortisol (Fig 4A).

Additionally, Area under the Curve (AUC) values were calculated using the formulas proposed by Pruessner *et al*. [47]. In line with the aforementioned results, one-way ANOVAs revealed no significant group differences, neither for the area under the curve with respect to ground (AUCg; *F*(1, 25) = 1.306, *p* = 0.264) nor for the area under the curve with respect to increase (AUCi; *F*(1, 25) = 0.059, *p* = 0.810; Fig 4B).

### Salivary alpha amylase

**Fig 5:**
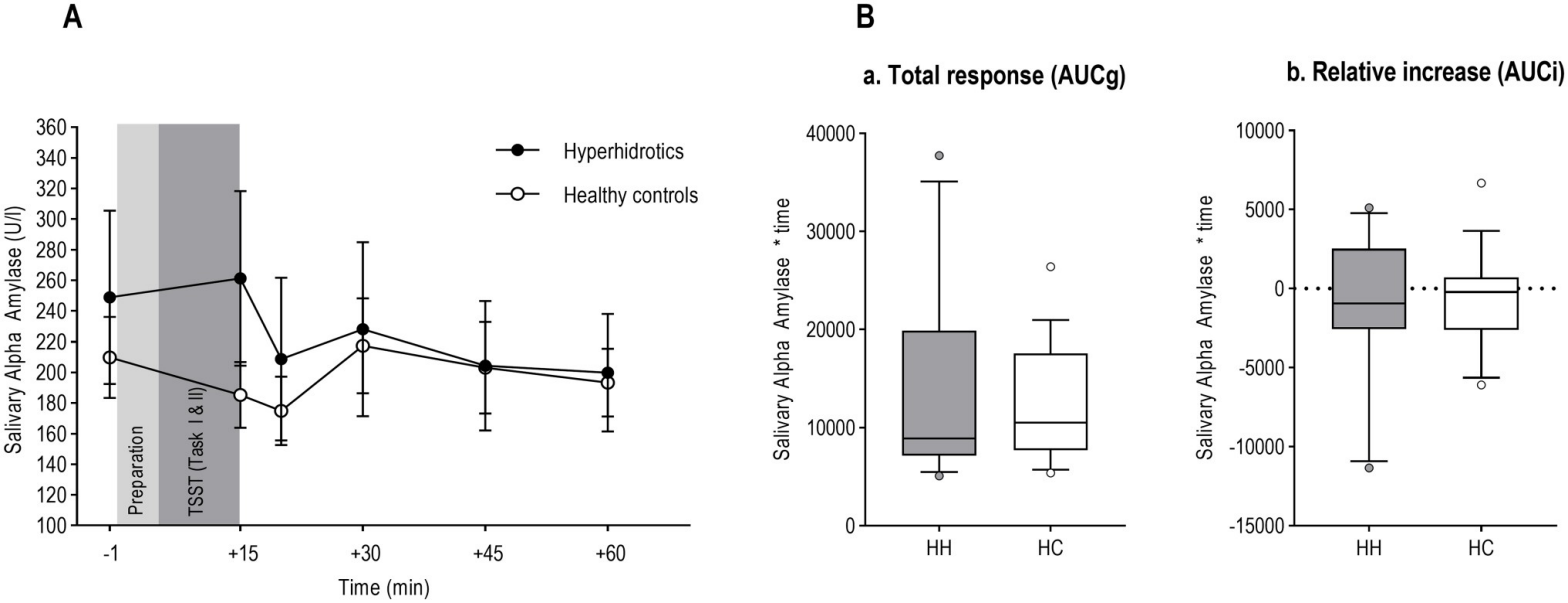
Analyses of salivary alpha amylase. (A) Salivary alpha amylase levels (U/l) in response to the stress condition over time. Preparation and TSST (Task I & II) represent the respective phases of the TSST. Error bars denote standard errors. (B) Boxplots for concentration of alpha amylase in response to the stressor for patients with PFH (HH) and healthy controls (HC). (a) Area under the Curve with respect to ground (AUCg). (b) Area under the Curve with respect to increase (AUCi). Whiskers denote 10^th^ and 90^th^ percentile.

Similar to cortisol, a 2 (PFH vs. healthy controls) x 6 (time) repeated-measures ANOVA was conducted to test for effects of the stressor on sAA (S3 Table). Contrary to all other measures, the analysis did not reveal a significant main effect of the factor time (*F*(2.950, 73.740) = 2.492, *ε* = 0.590, *p* = 0.068, *η*_*p*_^*2*^ = 0.091, 95% CI [160.590; 261.928]), indicating that sAA did not increase in response to the stressor. As with cortisol, the group x time interaction effect was also non-significant (*F*(2.950, 73.740) = 1.943, *ε* = 0.590, *p* = 0.131, Fig 5A).

AUC values were calculated for sAA using the formulas by 47 [47]. In line with the previously mentioned findings, one-way ANOVAs revealed no differences in AUCg values (*F*(1, 25) = 0.266, *p* = 0.611), or AUCi values (*F*(1, 25) = 0.287, *p* = 0.597) between patients with PFH and healthy controls (Fig 5B).

### Heart rate

**Fig 6:**
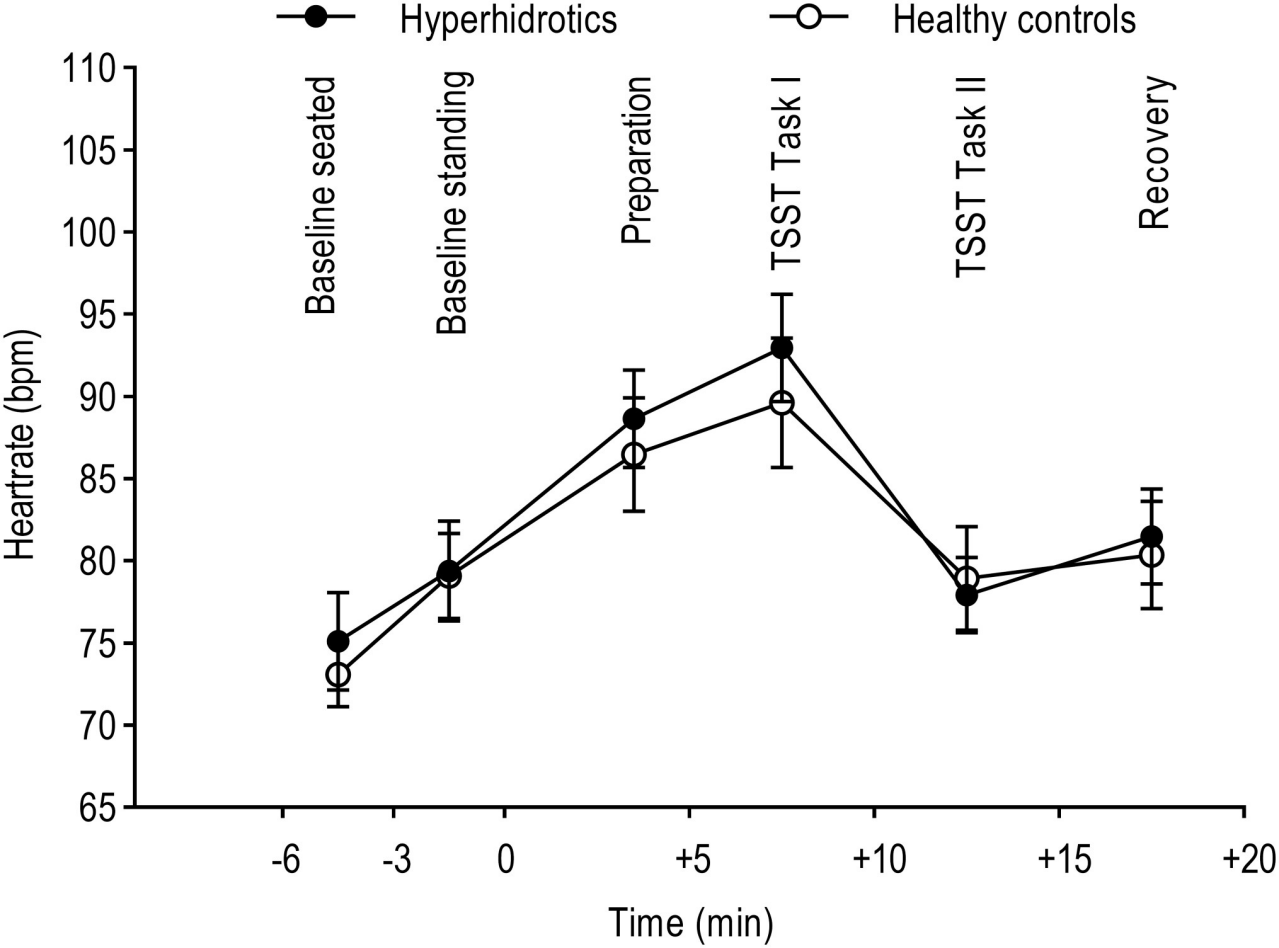
Analyses of heart rate. Mean values of heartrate (bpm) during six crucial phases of the TSST in virtual reality. Data missing from two healthy participants. Error bars denote standard errors.

As an additional indicator of SNS reactivity, we analysed mean values of heart rate (HR) in a 2 (PFH vs. healthy controls) x 6 (time) repeated-measures ANOVA. Prior to analysis, two healthy controls had to be excluded due to missing data. The time points refer to six crucial phases of the TSST paradigm, consisting of 3–5 minute intervals each: Baseline measurements prior to the TSST (seated and standing), preparation phase for the first task of the TSST, TSST Task I (simulated job interview), TSST Task II (arithmetic task), and a recovery phase after the TSST (S4 Table). The analysis revealed a significant main effect of the factor time (*F*(2.960, 68.075) = 45.351, *ε* = 0.592, *p* < 0.001, *η*_*p*_^*2*^ = 0.664, 95% CI [77.746; 86.101]) showing an increasing HR with its peak during the first task of the TSST (interview), but a non-significant group x time interaction effect (*F*(2.960, 68.075) = 0.654, *ε* = 0.592, *p* = 0.581; see Fig 6). This indicates an increase in heart rate measures in response to the stressor, which appeared to be similar between PFH patients and healthy controls.

### Axillary perspiration

**Fig 7:**
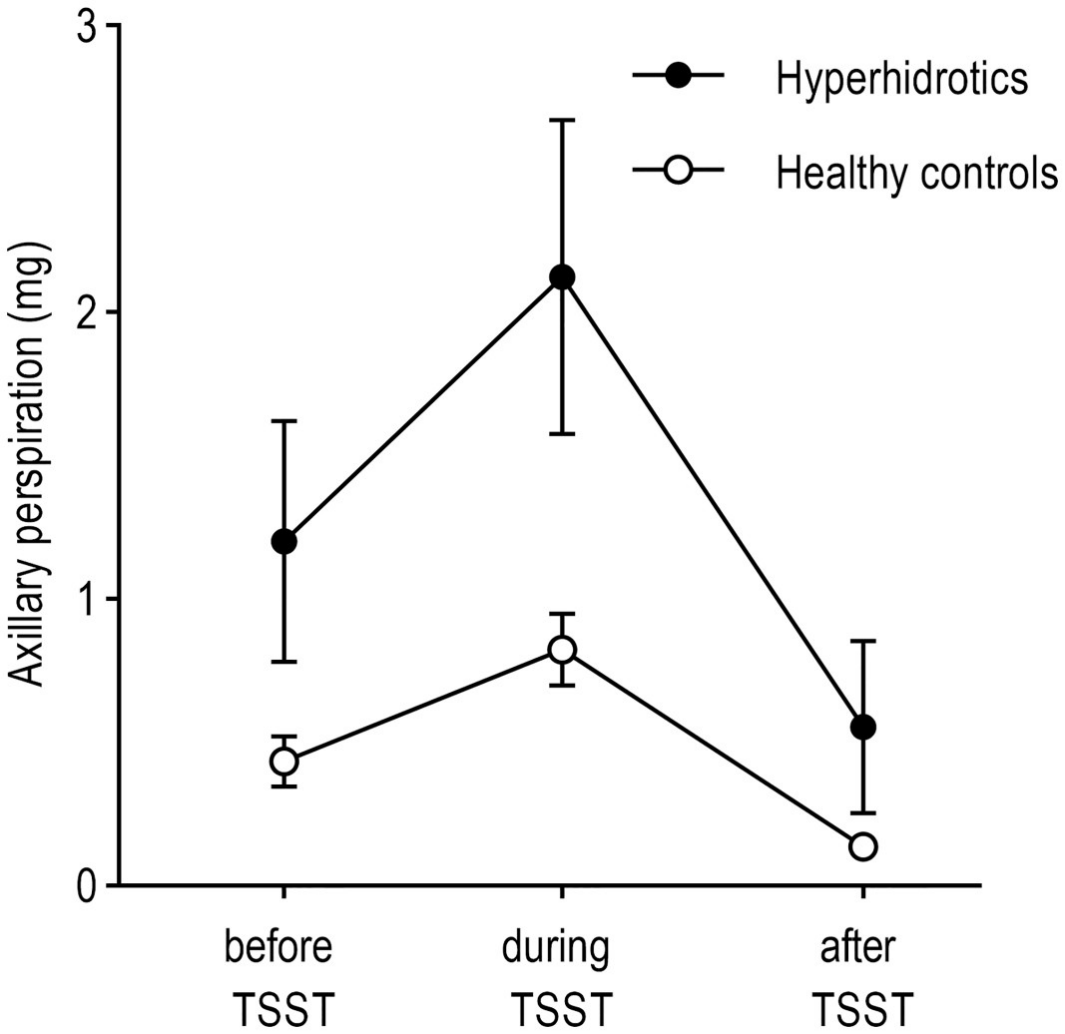
Analyses of axillary perspiration. Axillary perspiration (in mg) before, during, and after the TSST. Error bars denote standard errors.

For analysis of axillary perspiration during the experiment, we conducted a 2 (PFH vs. healthy controls) x 3 (time) repeated-measures ANOVA. Measures of axillary sweat secretion were taken before, during and after the TSST-VR during a time period of 15 minutes each (S5 Table). This ANOVA revealed a significant main effect of the factor time (*F*(1.754, 43.843) = 40.336, *ε* = 0.877, *p* < 0.001, *η*_*p*_^*2*^ = 0.617, 95% CI [0.524; 1.233]), indicating a significant increase in axillary perspiration during the TSST-VR. Additionally, a significant group x time interaction effect was found (*F*(1.754, 43.843) = 6.183, *ε* = 0.877, *p* = 0.006, *η*_*p*_^*2*^ = 0.198, 95% CI [0.524; 1.233]), indicating that PFH patients showed a significantly higher increase of axillary perspiration (see Fig 7). Due to violation of normal distribution and homoscedasticity (Kolmogorov-Smirnov *p* < 0.05, Levene-Test *p* < 0.01, respectively), Mann Whitney *U* tests were conducted to further analyse group differences at each time point. These analyses revealed significantly higher axillary perspiration of PFH patients during (*U* = 38.5, *Z* = -2.443, *p* = 0.015), but not before or after the TSST-VR (*U* = 55.5, *Z* = -1.604, *p* = 0.11; *U* = 69.5, *Z* = -0.914, *p* = 0.361, respectively), compared to healthy controls.

## Discussion

PFH puts a high psychological strain on affected people as it impairs life quality and wellbeing of the patients. Especially in stressful situations with e.g. social evaluation, PFH patients react with increased sweating resulting in stress and even more sweating [3].

In our pilot study, we investigated the stress reactivity in subjects diagnosed with primary hyperhidrosis. Both groups, PFH patients and healthy control subjects, showed expected responses to the TSST-VR leading to increased subjective stress, cortisol, heart rate as well as highest perspiration during the test. Further, we found that subjective stress, and cortisol levels as parameter of HPA axis reactivity as well as heart rate and alpha amylase did not differ significantly between both groups. However, in the patients with PFH subjects, we determined higher sAA as well as cortisol amounts in response to the stressor and before the tests on a descriptive level. In line with our expectation, we measured higher sweat secretion in PFH patients over the whole experimental setting and revealed a significant increase of perspiration during the TSS-VR in comparison to the healthy control subjects.

Although patients with PFH report significantly higher chronic stress, depressive symptoms, anxiety and decrease life quality [14,19,48,49], research on stress reactivity with focus on involvement of HPA axis and SAM activation is still missing for this disorder. Only one previous study investigated the cortisol morning profile and did not find a significant difference between patients with PFH and healthy control subjects, while the PFH patients reported increased depressive symptoms and more chronic stress compared to the control group [19]. Accordingly, the activity of the HPA axis appears adaptive, even if the perception and interpretation tendencies regarding a stimulus seem to be maladaptive in PFH patients. Due to negative experiences, specific expectations, assumptions and cognitions towards social-interactions, the exposure to a socially-evaluative situation could cause a stronger objective and subjective stress response in PFH patients. Therefore, we expected higher cortisol level in our patients with PFH after a challenging stress test. However, our results do not allow a final evaluation as the power to interpret non-significant findings was not high enough (Power (1-beta) = 37%). Psychosocial stress and sweating form a vicious cycle, in which stress leads to sweating, which in turn reinforces the self-perception of one’s own sweating as stressful [3]. Thus, ascertaining which of the two phenomena is at the basis of the cycle aggravated, posing a sort of chicken-and-egg problem [19]. If assuming a normal adaptability of the HPA axis in terms of cortisol in PFH patients, cortisol as a factor directly regulating stress-induced sweating might be excluded. A similar idea was presented by Harker [18], who wrote that no evidence suggests stress-induced HPA axis-associated glucocorticoids to activate or modulate sweat gland activity (albeit without presenting any actual experimental data).

PFH has been described related to an increased activity of the sympathetic nervous system [50,51]. sAA can be used as a marker of the SAM activation as well as an indicator of the dynamics of the stress response by calculating the AUCi [47,52–54]. In line with this, patients with PFH showed on a descriptive level higher sAA in our setting. However, careful interpretation is warranted as our findings have to be considered preliminary. Interestingly, we did not find differences in heart rate neither at baseline nor in response to the stress test. Similar findings have been reported during controlled respiration and handgrip exercise [55], and with short-term frequency domain power spectral analysis of heart rate variability [56,57]. Together with our preliminary findings, these provide further evidence that hyperhidrosis is a complex autonomic dysfunction rather than only a sympathetic overactivity, and that the parasympathetic system might be involved, too.

The link between sensual response of a stressor and sweat response is not fully understood, but imaging technologies have helped to suggest core roles of certain brain areas in this mechanism. Mental stress as elicited by mental calculation used in our TSST-VR paradigm triggers sweating on glabrous (non-apocrine) skin, a phenomenon termed mental sweating response (MSR). In combination with electroencephalographic tracing methods, Homma and colleagues [58,59] located stress-induced activation prior to measurable MSR first in the inferior frontal gyrus, followed by the lateral hippocampus in some patients, and the amygdala in others. The role of the amygdala was further substantiated by Asahina and colleagues [60], who demonstrated that a patient suffering from limbic encephalitis, comprising bilateral restricted amygdala lesions, did not sweat in response to sympathetic stimuli. As subjects responded neither to thermal nor tactile stimuli either, the amygdala and the limbic system can be suggested as key components in sweat secretion beyond emotional and mental stress stimuli, reinforcing the notion of a stronger interrelation between thermoregulatory and non-thermoregulatory perspiration. In our study, we found significant higher perspiration and a significant increase of sweat secretion in PFH patients compared to healthy control subjects in response to the TSST-VR. This perspiration might be induced by cholinergically activated neurons innervating eccrine sweat glands [50] and in addition by noradrenergic sympathetic vasoconstrictor nerves in cutaneous arterioles in glabrous skin that may simultaneously innervate sweat glands [18,61]. Another study supporting these assumption showed that the responsiveness of skin sympathetic nerve activities to mental stimuli was three to eight times as much enhanced in the tibial nerve as compared with the control group [16].

Apart from the influence of catecholamines suggested above, humoral control over sweat gland activation is thought to be a minor factor. Increased levels of sex steroid hormones were found coinciding with psychosocial stress [62], although no direct pathway has been proposed [18]. Additionally, axillary stress sweating is not prominent until puberty, thus a potential hormonal priming via androgens and estrogenes for specific sweat responses has been discussed [63–65]. In our study, we controlled for gender effects to rule out such influences and notably, testosterone has been found to increase apocrine gland size in both sexes [66], which might also allow to neglect gender as a variable in our preliminary investigation.

The present pilot study has some limitations: The sample of our pilot study is not representative for all patients with PFH because many criteria had to be fulfilled to fit the standardized procedures of the VR-TSST setting. As such, PFH patients suffering from depression and social withdrawal have been excluded during the recruitment process. However, this procedure was necessary to draw conclusions on the stress reaction in hyperhidrotics without major bias of other comorbidities or confounding factors. Therefore, the sample size was relatively small, with 11 diagnosed PFH patients versus 16 healthy controls, and the data is in need of replication in a study with more participants and additional control groups. The required sample size for such a study would include 108 participants in total, if considering 2 groups (participants with or without PFH) and two conditions (stress vs non-stress) in an ANOVA with repeated measurement between subjects design with an effect size f = 0.25 and a power (1-beta) = 0.80. An interesting adaptation would include a more detailed investigation of axillary *vs*. palmar/plantar PFH, which would require approximately 30 participants more. For such a setting, the TSST might be a good method for stress induction. We used the virtual form of the TSST [34,35], in which the participants had to interact with programmed entities instead of unapproachable human judges. Although, one might argue that the participants will be able to envision that they are not actually performing in front of real human beings, our and previous studies showed that the participants still experience social evaluative stress in the presence of virtual agents, as indicated by the subjective and endocrine reactions [34,35,42,67]. Another factor that might have influenced the subjective feelings as well as stress-related parameters was that the management of the VR paradigm required the presence of the experimenter at all times. The knowledge that there still was another person in the room, while one was performing the job interview and mental arithmetic tasks might have added an additional layer of social evaluation. Further and in addition to the standard protocol [34,35,67], in our study the measurement of sweat secretion required an interaction of the experimenter with the participants before, during and after the TSST-VR, which might have affected the outcome variables increasing anticipation effects. However, as both investigated groups were treated identically, possible differences in the perception of this interaction might be subjective and evaluated with the visual analogue scale in our study. In further studies, the technical improvements in the field of virtual reality including better graphics, higher resolution and more frames per second as well as more adaptive environments using e. g. electrodermal activity sensor systems [31] might be used for the investigation of stress reactivity of patients with PFH in even more realistic settings. As such, scenarios that more closely resembling typical adverse situations for most hyperhidrotics such as dating situations or interactions with superiors could be examined in the future.

In our pilot study, the parameters of endocrine stress responses were analysed using salivary measures of cortisol and alpha amylase. These measures have been proven valid indicators of HPA-axis and catecholaminergic stress reactivity [53,68]. However, direct measures of ACTH, cortisol and catecholamines in plasma would have possibly been more sensitive for the assessment of parameters important for the stress-dependent regulation of sweat secretion in patients with PFH and might be included in more extensive studies.

## Conclusion

In conclusion, we investigated the stress reactivity in patients with PFH using a standardized stress test. As expected, but experimentally shown for the first time, patients with PFH showed higher perspiration with a significant peak increase during the socially evaluative stressor compared to healthy control subjects. Subjective stress levels and endocrine parameters such as cortisol as well as heart rate were significantly increased after stress exposure but did not differ between both groups. On a descriptive level, slightly higher cortisol and sAA levels have been observed in patients with PFH. Although our data are in need of replication, sAA might be an interesting marker for further studies on stress induced reactivity of the sympathetic system and its possible connection to emotional sweating in patients with PFH.

## Supporting information

S1 Fig Screenshots from the virtual environment. (PPTX)

S1 Table Sample characteristics. (DOCX)

S2 Table Group differences in objective stress response (cortisol). (DOCX)

S3 Table Group differences in objective stress response (alpha amylase). (DOCX)

S4 Table Group differences across time points for objective stress response (heartrate). (DOCX)

S5 Table Group differences in axillary perspiration. (DOCX)

S6 Table Group differences in subjective stress response (VAS). (DOCX)
