# Supplementary material for: Real sweating in a virtual stress environment: Investigation of the stress reactivity in people with primary focal hyperhidrosis
Source: PLoS One. 2022 Aug 2;17(8):e0272247. doi: 10.1371/journal.pone.0272247 (PMC9345359; doi:10.1371/journal.pone.0272247)
Supplement: S1 Fig — (PPTX) [file pone.0272247.s001.pptx]

## Slide 1
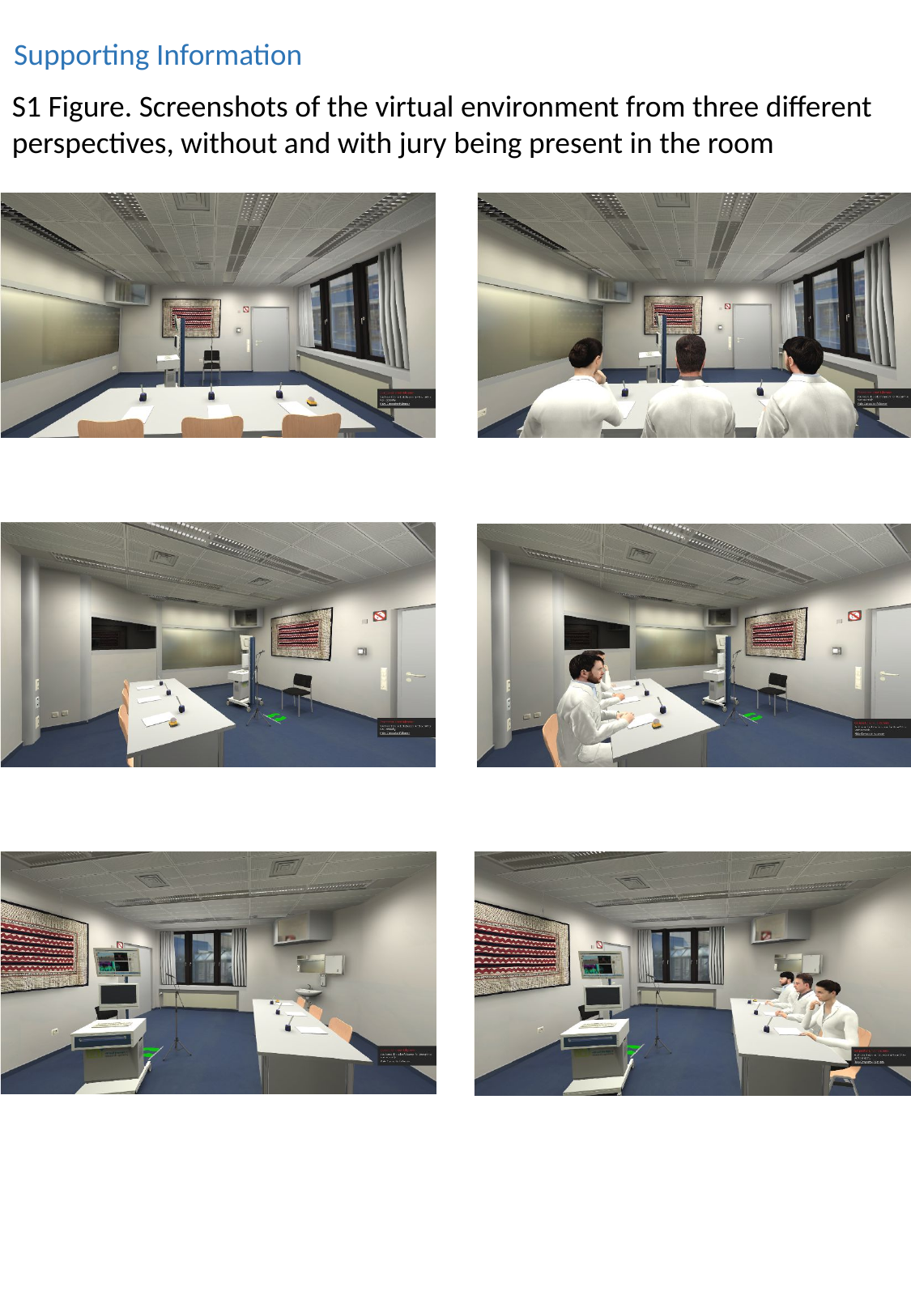

Supporting Information
S1 Figure. Screenshots of the virtual environment from three different perspectives, without and with jury being present in the room
