# Supplementary material for: Real sweating in a virtual stress environment: Investigation of the stress reactivity in people with primary focal hyperhidrosis
Source: PLoS One. 2022 Aug 2;17(8):e0272247. doi: 10.1371/journal.pone.0272247 (PMC9345359; doi:10.1371/journal.pone.0272247)
Supplement: S1 Table — (DOCX) [file pone.0272247.s002.docx]

# Supporting Information

**S1 Table.** Sample Characteristics.

|  | PFH patients  (*n* = 11) | | Healthy controls  (*n* = 16) | |  |
| --- | --- | --- | --- | --- | --- |
| Variable | *M (SD)* | *n* (%) | *M (SD)* | *n* (%) | *p* |
| Age | 36.1 (17.6) |  | 33.3 (16.1) |  | 0.675 |
| Sex (female)^a^ |  | 4 (36.4) |  | 7 (43.8) | 0.701 |
| Weight in kg | 72.6 (17.4) |  | 72.3 (16.1) |  | 0.961 |
| Height in cm | 174.6 (11.0) |  | 174.4 (10.2) |  | 0.962 |
| BMI | 23.5 (3.4) |  | 23.6 (3.9) |  | 0.965 |

**Note.** ^a^analysed with χ^2^-test, *p* < 0.05*.
