# Supplementary material for: Real sweating in a virtual stress environment: Investigation of the stress reactivity in people with primary focal hyperhidrosis
Source: PLoS One. 2022 Aug 2;17(8):e0272247. doi: 10.1371/journal.pone.0272247 (PMC9345359; doi:10.1371/journal.pone.0272247)
Supplement: S2 Table — (DOCX) [file pone.0272247.s003.docx]

# Supporting Information

**S2 Table.** Group differences in objective stress response (cortisol).

|  | PFH patients  (*n* = 11) | Healthy controls  (*n* = 16) |  |  |  |
| --- | --- | --- | --- | --- | --- |
| Time points^a^ | *M (SD)* | *M (SD)* | *U* | Z | *p* |
| TSST-VR -1 | 4.95 (6.07) | 3.41 (1.87) | 76.000 | -0.592 | 0.554 |
| TSST-VR +15 | 5.97 (6.70) | 3.56 (2.29) | 70.000 | -0.888 | 0.374 |
| TSST-VR +20 | 7.39 (7.57) | 4.42 (3.37) | 62.000 | -1.283 | 0.199 |
| TSST-VR +30 | 6.59 (6.06) | 4.68 (3.94) | 71.000 | -0.839 | 0.402 |
| TSST-VR +45 | 4.76 (3.87) | 3.72 (3.08) | 73.000 | -0.740 | 0.459 |
| TSST-VR +60 | 3.93 (2.99) | 2.84 (2.22) | 63.000 | -1.234 | 0.217 |

**Note.** ^a^time points in minutes. *p* = two-tailed asymptotic significance level, *p* < 0.05*.
