# Supplementary material for: Real sweating in a virtual stress environment: Investigation of the stress reactivity in people with primary focal hyperhidrosis
Source: PLoS One. 2022 Aug 2;17(8):e0272247. doi: 10.1371/journal.pone.0272247 (PMC9345359; doi:10.1371/journal.pone.0272247)
Supplement: S3 Table — (DOCX) [file pone.0272247.s004.docx]

# Supporting Information

**S3 Table.** Group differences in objective stress response (alpha amylase).

|  | PFH patients  (*n* = 11) | Healthy controls  (*n* = 16) |  |  |  |
| --- | --- | --- | --- | --- | --- |
| Time points^a^ | *M (SD)* | *M (SD)* | *U* | *Z* | *p* |
| TSST-VR -1 | 249.060 (187.75) | 209.760 (106.19) | 85.000 | -0.l48 | 0.882 |
| TSST-VR +15 | 261.277 (189.08) | 185.178 (86.03) | 69.000 | -0.938 | 0.348 |
| TSST-VR +20 | 208.598 (176.51) | 174.834 (89.03) | 86.000 | -0.099 | 0.921 |
| TSST-VR +30 | 228.263 (188.62) | 217.688 (123.92) | 78.000 | -0.493 | 0.622 |
| TSST-VR +45 | 204.337 (139.99) | 203.103 (119.75) | 83.000 | -0.247 | 0.805 |
| TSST-VR +60 | 199.838 (127.56) | 193.168 (88.72) | 85.000 | -0.148 | 0.882 |

**Note.** ^a^ time points in minutes. *p* = two-tailed asymptotic significance level, *p* < 0.05*.
