# Supplementary material for: Real sweating in a virtual stress environment: Investigation of the stress reactivity in people with primary focal hyperhidrosis
Source: PLoS One. 2022 Aug 2;17(8):e0272247. doi: 10.1371/journal.pone.0272247 (PMC9345359; doi:10.1371/journal.pone.0272247)
Supplement: S4 Table — (DOCX) [file pone.0272247.s005.docx]

# Supporting Information

**S4 Table.** Group differences across time points for objective stress response (heartrate).

|  | PFH patients  (*n* = 11) | Healthy controls  (*n* = 14^a^) |  |  |  |
| --- | --- | --- | --- | --- | --- |
| Time points | *M (SD)* | *M (SD)* | *T* | *df* | *p* |
| Baseline_seated | 75.12 (9.87) | 73.10 (7.33) | -0.590 | 23 | 0.561 |
| Baseline_standing | 79.39 (10.14) | 79.07 (9.70) | -0.081 | 23 | 0.936 |
| TSST-VR_preparation | 88.63 (9.88) | 86.49 (12.94) | -0.456 | 23 | 0.653 |
| TSST-VR_speech | 92.95 (10.84) | 89.63 (14.83) | -0.622 | 23 | 0.540 |
| TSST-VR_calculate | 77.93 (7.65) | 78.93 (11.74) | 0.244 | 23 | 0.809 |
| TSST-VR_recovery | 81.49 (9.67) | 80.36 (12.27) | -0.251 | 23 | 0.804 |

**Note.** ^a^ data from two healthy participants missing, p < 0.05*.
