# Supplementary material for: Real sweating in a virtual stress environment: Investigation of the stress reactivity in people with primary focal hyperhidrosis
Source: PLoS One. 2022 Aug 2;17(8):e0272247. doi: 10.1371/journal.pone.0272247 (PMC9345359; doi:10.1371/journal.pone.0272247)
Supplement: S5 Table — (DOCX) [file pone.0272247.s006.docx]

# Supporting Information

**S5 Table.** Group differences in axillary perspiration.

|  | PFH patients  (*n* = 11) | Healthy controls  (*n* = 16) |  |  |  |
| --- | --- | --- | --- | --- | --- |
| Time points | *M (SD)* | *M (SD)* | *U* | *Z* | *p* |
| Before TSST-VR | 1.20 (1.40) | 0.43 (0.35) | 55.500 | -1.604 | 0.109 |
| During TSST-VR | 2.12 (1.82) | 0.82 (0.50) | 38.500 | -2.443 | 0.015* |
| After TSST-VR | 0.55 (1.00) | 0.14 (0.16) | 69.500 | -0.914 | 0.361 |

**Note.** *p* = one-tailed exact significance level, *p* < 0.05*.
