# Supplementary material for: Real sweating in a virtual stress environment: Investigation of the stress reactivity in people with primary focal hyperhidrosis
Source: PLoS One. 2022 Aug 2;17(8):e0272247. doi: 10.1371/journal.pone.0272247 (PMC9345359; doi:10.1371/journal.pone.0272247)
Supplement: S6 Table — (DOCX) [file pone.0272247.s007.docx]

# Supporting Information

**S6 Table.** Group differences in subjective stress response (VAS).

|  | PFH patients  (*n* = 11) | Healthy controls  (*n* = 16) |  |  |  |
| --- | --- | --- | --- | --- | --- |
| Time points^a^ | *M (SD)* | *M (SD)* | *U* | Z | *p* |
| TSST-VR -35^b^ | 23.70 (21.67) | 26.16 (19.75) | 69.000 | -0.580 | 0.562 |
| TSST-VR -20 | 16.86 (16.49) | 22.85 (21.46) | 72.000 | -0.790 | 0.430 |
| TSST-VR +15 | 35.73 (21.89) | 35.81 (26.74) | 86.500 | -0.074 | 0.941 |
| TSST-VR +45 | 13.77 (14.51) | 14.22 (11.55) | 83.500 | -0.222 | 0.824 |
| TSST-VR +60 | 11.27 (11.25) | 9.41 (8.75) | 82.500 | -0.273 | 0.785 |

**Note.** ^a^time points in minutes. ^b^data missing from one healthy participant. *p* = one-tailed exact significance level, *p* < 0.05*. VAS_diff_ (VAS3-VAS2): *U* = 68.0, Z = -0.988, *p* = 0.323.
